# Exploring time-varying impact of world pandemic uncertainty on China's commodity prices using TVP-SVAR-SV model

**DOI:** 10.3389/fpubh.2022.950010

**Published:** 2022-08-15

**Authors:** Qiang Cao, Xiu-qi Yang, Hu Chen, Wenmei Yu

**Affiliations:** Anhui University of Finance and Economics, Bengbu, China

**Keywords:** world pandemic uncertainty, China's commodity prices, TVP-SVAR-SV model, time-varying, emerging economies/countries

## Abstract

Since the outbreak of the COVID-19 pandemic, a growing body of literature has focused on the impact of the uncertainty of the world pandemic (WPU) on commodity prices. Using the quarterly data from the first quarter of 2008 to the second quarter of 2020, we run the TVP-SVAR-SV model to study the time-varying impact of WPU on China's commodity prices. Specifically, we select minerals, non-ferrous metals, energy and steel commodities for a categorical comparison and measure the impact of WPU accordingly. The findings are as follows. First, WPU has a significant time-varying impact on China's commodity prices, and the short-term effect is greater than the long-term effect. Second, compared with the global financial crisis in the fourth quarter of 2008 and China's stock market crash in the second quarter of 2015, WPU had a greatest impact on Chinese commodity prices during the COVID-19 pandemic event in the fourth quarter of 2019. Third, significant differences exist in the impact of WPU on the four major commodity prices. Among them, WPU has the largest time-varying impact on the price of minerals but the smallest time-varying impact on that of steel.

## Introduction

World pandemic uncertainty (WPU) refers to the economic uncertainty triggered by the outbreak of world pandemics and other diseases. It often has a serious negative impact on the global real economy and financial markets. The economic uncertainty caused by the COVID-19 pandemic, for example, is estimated to be much higher than by the previous pandemics (1), paralyzing the global real economic activities (2, 3). In general, WPU leads to stagnation or recession of economic development, resulting in a decline in total demand and the depression in financial market (4–6). During the period of COVID-19 pandemic, the global crude oil market and financial market experienced a huge slump. The US stock market experienced four circuit breakers in March 2020. The Chinese stock market also experienced panic selling, leading to the turmoil in the stock market.

The systemic risk of the financial market may come from the commodity market, because commodities have both commodity and financial attributes. Under the impact of WPU, investors choose to regard commodities as high-quality hedging tools (7). The financial attributes of commodities can further break down to risk premium and futures investment attributes. Risk premium refers to the premium compensation of systematic risk, while futures investment is a financial attribute in the general sense. During the crisis, the systemic risk of the commodity market is small in the short term, but with the passage of time, the systemic risk accumulates and the risk premium compensation required by investors gradually becomes larger.

The WPU has an impact on global commodity prices (8, 9). In this paper, we focus instead on the impact of WPU on Chinese commodity prices. Chinese commodities cover a total of 26 commodities in 9 categories (i.e., minerals, non-ferrous metals, energy, steel, rubber, agriculture, livestock, vegetable oil, and sugar), of which the first four categories are more easily exposed to the impact of WPU. Thereby, we sample these four commodity indexes. The reasons are as follows. First, since China joined the WTO in 2001, the import volume of minerals, non-ferrous metals, energy, and steel has been expanding at an alarming rate, accompanied by the exponential growth in external dependence. So far, the demand for minerals in China have accounted for more than 50% of the international market, steel 68%, non-ferrous metals such as aluminum 74%, energy such as crude oil 26%, coal 18% and natural gas 11%. Second, Chinese enterprises that mainly import these four categories of commodities are often with low industrial concentration and less likely to establish effective purchasing alliances. Therefore, their prices directly affect China's import and export trade as well as the country's economic growth. Third, during the spread of the pandemic, the prices of energy, non-ferrous metals, and minerals have undergone major changes. For example, a report in 2020 pointed out that the pandemic has led to resections in travel and a halt in production, both of which have greatly curbed the demand for energy such as oil and non-ferrous metals, resulting in greater fluctuations in prices of the two categories (10). At the same time, the closure of mines in major mineral exporting countries has also led to a decline in the supply of mineral commodities and caused price fluctuations of mineral commodities (9). Fourth, whether the price indice of these four major commodity show the same trend during the WPU is also related to the likilyhood of systemic risks and in turn affects investors' adjustment in portfolio strategies.

Besides, we use the CCPI to measure the changes in the commodity price. This is because the volatility of the four commodities price sub-indices in CCPI directly affects China's economic growth as these commodities are often imported by large volumes, showing strong oversea dependence and low industrial concentration. During the spread of WPU, these four categories are more likely to undergo drastic changes and show the similar trend, forecasting subsequent systemic financial risks.

The marginal contribution of this paper is three-fold. First, we construct a theoretical mechanism of WPU on China's commodity prices, in which the risk premium channel is related to the systematic risk compensation and explains the time-varying effect as the systematic risk accumulates over time. Second, we sample the price of China's commodities, and select minerals, non-ferrous metals, energy and steel to examine the differences in the WPU impact on the price of different commodity categories. Thirdly, we utilize the TVP-SVAR-SV model. This model allows us to test the time-varying effect in the short, medium and long term, and thus enables us to compare the impact of the three events, so as to assess whether the impact of this round of COVID-19 pandemic show more serious consequences than the global financial crisis in 2008 and the stock market crash in 2015.

## Related works

The world pandemic has an impact on both financial and commodity markets. The impact on the financial market, is mainly reflected in the volatility of asset prices in different financial markets, such as securities market (11, 12), foreign exchange market (13), gold market (14). In the commodity market, the impact is mainly on the financial and commodity attributes of a given commodity category, as the financial attribute of the commodity often interacts with the financial market, leading to systemic risks. For example, Borgards et al. (15) concluded that the rise of WPU leads to an overreaction to the commodity futures price, especially the price of energy commodity futures.

The previous literature on the relationship between WPU and commodity prices set the WPU impact either on a global scale [e.g., (16)] or at a regional level, such as the United States (17), Europe (18), G7 (19), and the BRICS (20). In the selection of commodity indices, these researchers tend to use the BCOM index, which is more suitable for studying the fluctuations in the global commodity prices [e.g., (21)]. However, when a specific country is concerned, it is preferable to use its domestic commodity price index. For instance, Lin and Xu (22) examined the Chinese commodity prices by adopting CCPI.

Since there are a wide range of commodity categories due to varied criteria, most researchers typically select a single broad commodity index, such as agricultural commodities (23–25), energy (16), metals (26), and precious metals (20, 27). Still a few compared the differences between several commodity price sub-indices. For instance, Bakas and Triantafyllou (8) chose crude oil and gold as the most representative of commodity categories for their research. Troster and Kublbock (9) asserted that among the sub-categories of commodities, energy, metals, minerals and precious metals are more vulnerable to the WPU, while agricultural products are less likely to be affected.

Judging from the findings, it is evident that when the changes in the price of the sub-indexed commodities go in different directions, system risks are less likely to happen as the counterbalance automactically hedges the risks. For instance, Bakas and Triantafyllou (8) research attempted to correlate WPU with gold and petrol and found that WPU had a negative impact on the price index of petrol but a positive one on that of gold.

However, the literature also suggest that systemic risks are more likely to surface when the fluctuations in several commodity price sub-indices share the same pattern. For instance, Wei et al. (28) found that the long-term impact of the pandemic on the prices of gold and crude oil goes in similar patterns. According to Azimli (29), during the spread of the COVID-19 pandemic, copper, iron, gold and engery markets served the role of hedging the risks overlowing from the global stock maket.

From the perspective of methodology, the literature on the relationship between WPU and commodity prices is mainly divided into high- and low-frequency data categories. The research methods in the high-frequency category mainly include wavelet analysis (30, 31) and spillover model (24). And in the low-frequency category, the methodology mainly includes VAR model (32), SVAR model (33), TVP-SVAR-SV model (34).

To sum up, the existing literature is flawed in three aspects. First, although most researchers recognize the financial attribute of commodities, few have analyzed the risk premium channel of their financial attribute, which makes WPU positively connected to the commodity prices. Second, the existing literature mainly focuses on developed countries such as the US, or blocs such as G7, BRICS and EU, lacking the samples from emerging market countries, especially China. Thirdly, the literature is mainly based on high-frequency data research, and lacks comparative studies on short-term, medium-term and long-term time-varying relationships across different events.

The rest of this paper is organized as follows: Section Theoretical framework and research hypothesis expounds the theoretical framework and research hypothesis, Section Methodologies and data description the data and method used in this paper, Section Empirical results the empirical analysis of the WPU impact on China's commodity prices, and Section Conclusion and policy implication the conclusion and policy implication.

## Theoretical framework and research hypothesis

Most of the literature suggests that the occurrence of economic uncertainty has an impact on commodity prices (35–37), and WPU as a measure of economic uncertainty associated with world pandemics, we argue that WPU also affects commodity prices.

**Figure 1:**
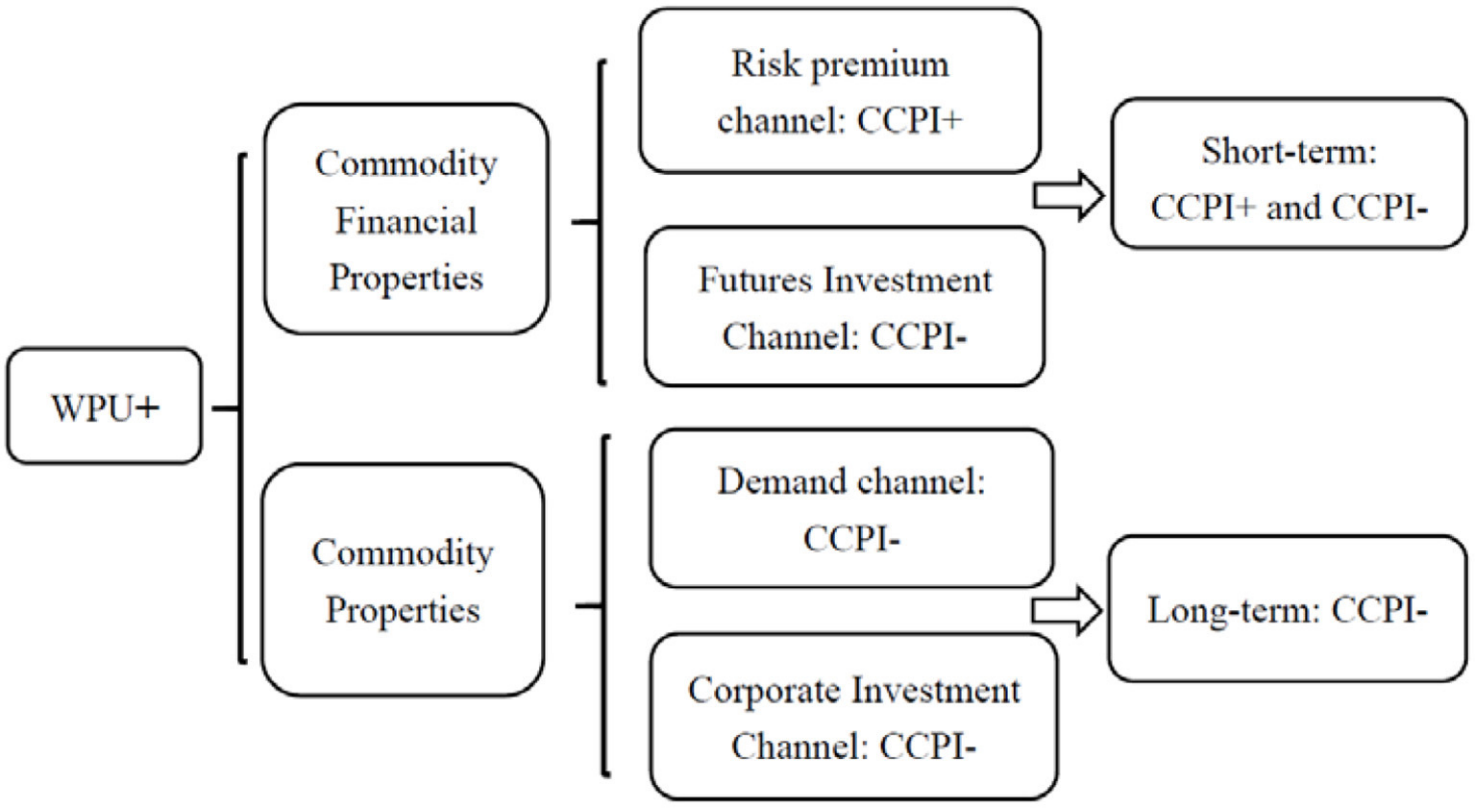
Theoretical transmission mechanism of WPU affecting commodity prices.

Generally, commodities have a dual attribute of both commodity and finance. The financial attributes of commodities are essentially financial factors at play, including risk premium channels and futures investment channels and the commodity attributes of commodities essentially real demand factors at play, including demand channels and corporate investment channels. Thus, we contend that WPU affects commodity prices by acting on both the commodity and financial channel. Figure 1 provides a diagram of the transmission mechanism by which the WPU affects commodity prices.

The WPU has an impact on commodity prices through the financial attributes of commodities. This is manifested as two channel effects from rising WPU: the risk premium channel (38) and the futures investment channel. On the one hand, WPU certainty affects commodity prices through the risk premium channel. Rising WPU leads to increased return risk for investors in commodity futures markets, which requires more risk premiums to be given to investors as risk compensation, which in turn leads to higher commodity prices. On the other hand, WPU can affect commodity prices through the futures investment channel. According to the risk aversion utility theory (39), a rise in WPU triggers a high level of negative investor sentiment and investors withdraw from commodity futures markets for hedging purposes to hedge their risks, and a decrease in investment in commodity futures leads to a decline in commodity futures prices, which in turn leads to a decline in commodity prices with financial attributes.

The WPU has an impact on commodity prices through the commodity properties of commodities, which manifests itself in two channel effects from rising WPU: the demand channel and the corporate investment channel. First, since demand factors are influential in affecting commodity prices (40), rising WPU triggers a decline in commodity demand through the demand channel, which in turn leads to a decline in commodity prices. Second, rising WPU leads to lower commodity prices through the corporate investment channel. On the one hand, real options theory (41) argues that firms postpone investment during periods of increased uncertainty, and firms expect to obtain more information about the future market to avoid possible risky losses, and the reduction in firm investment leads to lower commodity prices. On the other hand, financial friction theory suggests that increased uncertainty leads to increased financial frictions, and increased financial frictions lead to increased financing costs and difficulty in financing for firms (42), and firms will reduce foreign investment to preserve their asset and liability profiles, which in turn leads to lower commodity prices.

In summary, the financial attributes of commodities come into play in the short term, with WPU positively influencing commodity prices through the risk premium channel and negatively influencing commodity prices through the futures investment channel. The commodity attributes of commodities play a role in the long run, and the WPU negatively affects commodity prices through the demand channel and the corporate investment channel. Moreover, the process of commodity marketization in China changes over time, triggering uncertainty shocks that have time-varying effects on commodity prices (34, 43). Accordingly, we propose Hypothesis 1:

**H1**. *WPU has a negative impact on China's commodity prices with a significant time-varying impact effect*.

Second, compared to a poor economic environment, commodity markets are more conducive to their own development in a better economic environment, with greater risk resilience and the ability to recover themselves after risk. The changes in the economic environment in which commodity markets operate in different periods of time also lead to differences in the ability of commodity markets to withstand WPU shocks, which in turn leads to differences in the impact of WPU on commodity prices. Accordingly, we propose Hypothesis 2:

**H2**. *WPU has different effects on China's commodity prices in different periods*.

Finally, the WPU affects commodity prices through factors such as investment and demand, but the impact of uncertainty on commodity prices is affected by the industry in which the commodity is located (44). Minerals, non-ferrous metals, energy and steel account for the highest proportion of China's commodity market. They have varied degrees of importance in production, sales and trade. Therefore, the WPU impact varies across these four categories of commodities. Accordingly, we propose Hypothesis 3:

**H3**. *The WPU has a differential impact on commodity prices in the minerals, non-ferrous metals, energy and steel*.

## Methodologies and data description

### Methodologies

The TVP-SVAR-SV model is an extension of the basic SVAR model by adding time-varying parameters. We first constructed the SVAR model with six variables, as detailed in Equation (1):


(1) $$\begin{array}{r} Ay_{t}=\beta_{0}+\overset{n}{\underset{i=1}{\sum }}\beta_{i}y_{t-i}+\varepsilon_{t} \end{array}$$

Here $y_{t}={\left(WPU_{t},GDP_{t},CPI_{t},IR_{t},FIN_{t},CCPI_{t}\right)}^{\prime }$, where WPU denotes world pandemic uncertainty, GDP denotes China gross national product, CPI denotes China consumer price index, IR denotes interest rates, FIN denotes the degree of financialization of commodities indicator, and CCPI denotes China's commodity prices. β_0_ and β_*i*_ are 6 × 6 the coefficient matrices, with ε_*t*_ the structural shock vectors. We assume that the matrix *A* is invertible, so we substitute *A*^−1^ into Equation (1) to generate the reduced form of the VAR model:


(2) $$\begin{array}{r} y_{t}=A^{-1}\beta_{0}+A^{-1}{\overset{n}{\underset{i=1}{\sum }}\beta_{i}y_{t-i}+A^{-1}\varepsilon_{t}=\phi }_{0}\\ +\overset{n}{\underset{i=1}{\sum }}\phi_{i}y_{t-i}+e_{t} \end{array}$$

where, *e*_*t*_ is the perturbation term and $e_{t}=A^{\text{-1}}\varepsilon_{t}$. Here, to better identify the SVAR model, we impose constraints on *A*^−1^ for the following reasons. Firstly, WPU is the initial shock and thus is not affected by other factors. Secondly, according to the real cycle theory, long-run supply shocks are only affected by themselves. Besides, according to the monocentric view, money supply is only affected by both demand and supply shocks in the long run, and thus inflation is only affected by demand and supply shocks. Finally, low interest rates and high money supply are exogenous factors for the financialization of commodity prices, and the increase in financialization will also have an impact on commodity prices. Based on the above analysis, *e*_*t*_, the error in simplified form is expressed as Equation (3).


(3) $$\begin{array}{r} e_{t}=\left(\begin{array}{c} e_{t}^{WPU}\\ e_{t}^{GDP}\\ e_{t}^{CPI}\\ e_{t}^{IR}\\ e_{t}^{FIN}\\ e_{t}^{CCPI} \end{array}\right)=\left(\begin{array}{cccccc} \alpha_{11} & 0 & 0 & 0 & 0 & 0\\ \alpha_{21} & \alpha_{22} & 0 & 0 & 0 & 0\\ \alpha_{31} & \alpha_{32} & \alpha_{33} & 0 & 0 & 0\\ \alpha_{41} & \alpha_{42} & \alpha_{43} & \alpha_{44} & 0 & 0\\ \alpha_{51} & \alpha_{52} & \alpha_{53} & \alpha_{54} & \alpha_{55} & 0\\ \alpha_{61} & \alpha_{62} & \alpha_{63} & \alpha_{64} & \alpha_{65} & \alpha_{66} \end{array}\right)\times \left(\begin{array}{c} \varepsilon_{t}^{WPU}\\ \varepsilon_{t}^{GDP}\\ \varepsilon_{t}^{CPI}\\ \varepsilon_{t}^{IR}\\ \varepsilon_{t}^{FIN}\\ \varepsilon_{t}^{CCPI} \end{array}\right) \end{array}$$

On the basis of the SVAR model, we further construct the TVP-SVAR-SV model that enable us to fully capture the time-varying effects of WPU on commodity prices at various stages by setting time parameters in the SVAR model. According to Nakajima (45) and Primiceri (46), Equation (1) can be written in the form of Equation (4).


(4) $$\begin{array}{r} y_{t}=x_{t}\beta +A^{-1}\sum \varepsilon_{t} \end{array}$$

Where β is the dimensional vector of (36*i*+6), $X_{t}=I_{i}⊗\left({y_{t-1}}^{\prime },\cdots \;,{y_{t-i}}^{\prime }\right)$ and ∑ the 7 × 7 dimensional diagonal matrix and the diagonal [σ_1_, σ_2_, ⋯σ_6_]. We add the time factors to Equation (4) to derive the TVP-SVAR-SV model as


(5) $$\begin{array}{r} y_{t}=x_{t}\beta_{t}+{A_{t}}^{-1}\sum_{t}\varepsilon_{t} \end{array}$$

Equation (5) is the observed equation, and according to Primiceri (46) and Nakajima (45), the parameters are assumed to follow the following random walk process:


(6) $$\begin{array}{r} \begin{array}{c} \beta_{t+1}=\beta_{t}+u_{\beta t}\\ \alpha_{t+1}=\alpha_{t}+u_{\alpha t}\\ h_{t+1}=h_{t}+u_{ht} \end{array} \end{array}$$

Where $h_{t}={\left(h_{1t},h_{2t},h_{3t},h_{4t},h_{5t},h_{6t}\right)}^{\prime }$, $h_{jt}=\log\sigma_{jt}^{2},j=1,\ldots 6,t=s+1,\ldots n$.


(7) $$\begin{array}{r} \begin{array}{c} \beta_{s+1}\sim N\left(u_{\beta_{0}},\sum_{\beta_{0}}\right)\\ \alpha_{s+1}\sim N\left(u_{\alpha_{0}},\sum_{\alpha_{0}}\right)\\ h_{s+1}\sim N\left(u_{h_{0}},\sum_{h_{0}}\right) \end{array} \end{array}$$

The variance covariance matrix of this model is diagonal:


(8) $$\begin{array}{r} \left(\begin{array}{c} \varepsilon_{t}\\ u_{\beta t}\\ u_{\alpha t}\\ u_{ht} \end{array}\right)\sim N\left(0,\left(\begin{array}{cccc} I & 0 & 0 & 0\\ 0 & \sum_{\beta } & 0 & 0\\ 0 & 0 & \sum_{\alpha } & 0\\ 0 & 0 & 0 & \sum_{h} \end{array}\right)\right) \end{array}$$

where ∑_β_, ∑_α_, and ∑_*h*_ are assumed to be diagonal matrices.

### Data description

Our dataset includes the *China Commodity Price Index (CCPI), the WPU Index (WPUI), China Gross Domestic Product (GDP), Consumer Price Index (CPI), Interest Rates (IR) and the China Commodity Financialization Index (FIN)* from the first quarter of 2008 to the second quarter of 2020.

#### Explained variable

##### China commodity price index

Compared with the BCOM commodity index, which is widely used in the study of the global commodity market, we use the CCPI commodity index to classify commodities. Reasons are as follows. First, the research objects are different. The BCOM index is a global commodity price index and is mainly used to study the global commodity prices, as is seen in Bakas and Triantafyllou (8), but our focus is on the emerging market, so we use the CCPI index instead. Second, futures trading is different from spot trading. The BCOM index is based on futures trading prices, so it has both investment and speculative attributes, suitable for the research in developed countries. In the emerging markets such as China, commodity futures markets are underdeveloped, and it is hard to generate widely recognized futures prices. Therefore, it is not suitable to use the BCOM index. CCPI index, on the other hand, is the commodity spot database established by the China International Electronic Commerce Center, which emphasizes the spot transaction. Based on the price, the index is calculated using the weighted average method with June 2006 as the baseline period, covering a total of 26 commodities in 9 categories (minerals, non-ferrous metals, energy, steel, rubber, agricultural products, livestock, vegetable oil, and sugar). Third, the weights of the indicators in the index are different. The BCOM index emphasizes on the equalization of weights, and precious metals (such as gold) account for nearly 20%, which automatically invites frequent transactions for the purpose of investment or speculation. By contrast, the CCPI index add more weights to four categories: mineral products, non-ferrous metals, energy, and steel. Therefore, as compared to the construct of the BCOM index, the CCPI index is more representative of the fluctuation in the prices of commodity in China.

#### Explanatory variable

##### The WPU index (WPUI)^1^

To accurately measure world pandemic uncertainty, we select the WPU index measured by Ahir et al. (1), which measures the economic uncertainty induced by a world pandemic. The similar study includes Gozgor et al. (47).

#### Control variables

##### China gross domestic product

Changes in demand due to economic growth are an important factor affecting commodity prices (48), and we choose China gross domestic product (GDP) provided by the WIEGO statistical database to respond to changes in demand due to China's economic growth.

##### China consumer price index

We select the China Consumer Price Index (CPI) provided by the WIEGO statistical database as a proxy variable for inflation in China.

##### Interest rate

After the global financial crisis in 2008, monetary policy has gradually become an important factor influencing commodity prices (49), and we choose the 7-day weighted average interbank interest rate in China provided by the WIEGO statistical database as a proxy variable for China's monetary policy.

##### China commodity financialization index

We obtain the dynamic correlation coefficients between the China's commodity futures price index and the China SSE Composite Index to measure the financialization degree of China's commodities using the DCC-GARCH model based on Liu et al. (50), with data from the Flush database. In addition, we convert all data into quarterly data to ensure the uniformity of data frequency.

### Unit root test

We are using time series data, and the smoothness of the data is a prerequisite for the accuracy of the regression results. Therefore, we use the ADF test to check the smoothness of our time series data.

As can be seen from Table 1, only the variable CPI is smooth at the 1% significance level but the first-order differencing of all variables after are smooth at 1% significance level. Therefore, in this paper, the TVP-SVAR-SV model is constructed using the first-order differencing series.

**Table 1:** Unit root ADF test results.

	**Variable**	**ADF**	**1%**	**5%**	***P***	**Conclusion**
Original level	WPUI	1.104282	−3.57131	−2.92245	*P*≥0.05	Unstable
	CCPI	−1.98215	−3.57131	−2.92245	*P*≥0.05	Unstable
	GDP	−0.70088	−3.57445	−2.92378	*P*≥0.05	Unstable
	CPI	−3.60302	−3.57131	−2.92245	*P* ≤ 0.01	Stable
	IR	−2.23149	−3.54446	−2.92378	*P*≥0.05	Unstable
	FIN	−2.48594	−3.57131	−2.92245	*P*≥0.05	Unstable
First-order difference	wpui	−5.67866	−3.57445	−2.92378	*P* ≤ 0.001	Stable
	ccpi	−5.94458	−3.57445	−2.92378	*P* ≤ 0.001	Stable
	gdp	−10.311	−3.57445	−2.92378	*P* ≤ 0.001	Stable
	cpi	−6.40928	−3.58474	−2.92814	*P* ≤ 0.001	Stable
	ir	−12.7928	−3.57446	−2.92378	*P* ≤ 0.001	Stable
	fin	−8.97128	−3.57445	−2.92378	*P* ≤ 0.001	Stable

## Empirical results

### Estimation of selected parameters

According to Nakajima (45), we set the initial values: μ_*a*_0__ = μ_β_0__ = μ_*h*_0__ = 0, ∑_*a*_0__ = ∑_β_0__ = ∑_*h*_0__ = 10 × *I*, ${\left(\sum_{\beta }\right)}_{i}^{-2}\sim Gamma\left(20,10^{-4}\right)$, ${\left(\sum_{a}\right)}_{i}^{-2}\sim Gamma\left(4,10^{-4}\right)$, ${\left(\sum_{h}\right)}_{i}^{-2}\sim Gamma\left(4,10^{-4}\right)$. And we establish a TVP-SVAR-SV model in which the lag order is set to 1 based on Schwarz Criterion (SC) and the Hannan-Quinn information criterion (HQ). We use OxMetrics 6 to execute the MCMC algorithm on 10,000 samples and discard the first 1,000 samples to obtain valid samples for the posterior estimation of the model.

As shown in Table 2, the parameter estimation results of the MCMC simulation method show that the Geweke values of each parameter are <1.96, indicating that the null hypothesis that the results tend to be posteriori distributed cannot be rejected at the 5% confidence level. The maximum value of the invalidation factor does not exceed 159.48, indicating that at least 62 (10,000/159.48) irrelevant samples are generated during 10,000 iterations, which suggests that the samples generated during the iterations are valid.

**Table 2:** Estimation results of the selected parameters in the TVP-SVAR-SV mode.

**Parameters**	**Mean**	**St. dev**.	**95% interval**	**Geweke**	**Inef**.
(_Σ_β_)1_	0.0228	0.0026	(0.0184, 0.0286)	0.401	3.64
(_Σ_β_)2_	0.0227	0.0026	(0.0183, 0.0283)	0.826	3.69
(_Σ_α_)1_	0.1059	0.2764	(0.0428, 0.2255)	0.082	16.76
(_Σ_α_)2_	0.0845	0.0374	(0.0421, 0.1843)	0.001	29.08
(_Σ_*h*_)1_	1.9175	0.6418	(0.9324, 3.3939)	0.413	159.48
(_Σ_*h*_)2_	0.5409	0.2042	(0.2523, 1.0303)	0.77	75.85

Mean denotes posterior means; St. dev. denotes standard deviations; Inef. denotes the inefficiency factor.

**Figure 2:**
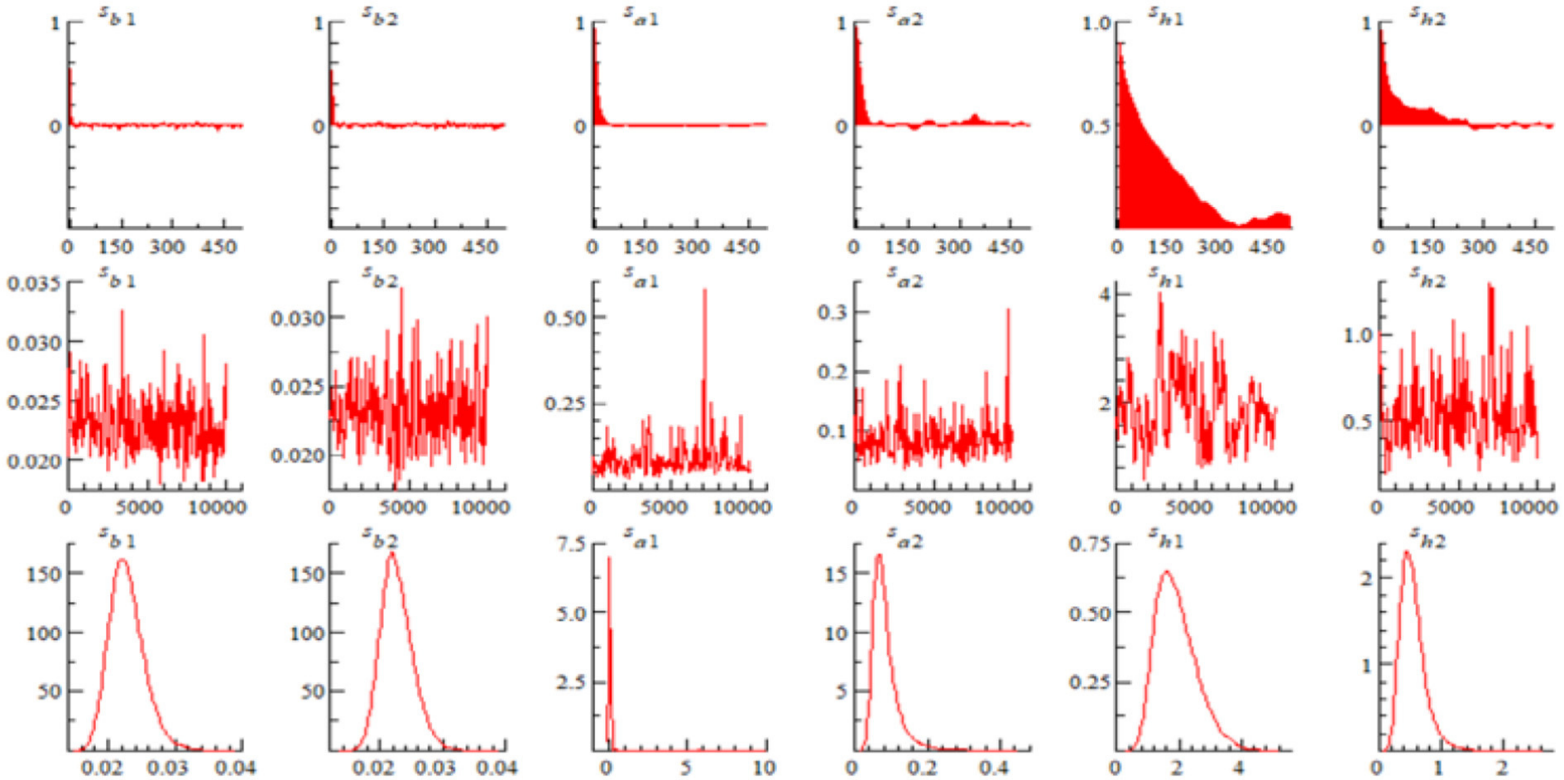
Sample autocorrelation, sample paths and posterior densities for selected parameters.

Figure 2 gives the sample autocorrelation plot, sample path and posterior density plot of the parameters. The results show that MCMC sampling is valid and the model estimation results are good.

### The time-varying effects of WPU on China's commodity prices

To investigate the time-varying impact of WPU on China's commodity prices, we use the TVP-SVAR-SV model to conduct equal-interval time-varying impulse responses with lags of 2, 4, and 6 periods, respectively, to characterize the impact of short-term, medium-term, and long-term WPU shocks on China's commodity prices.

**Figure 3:**
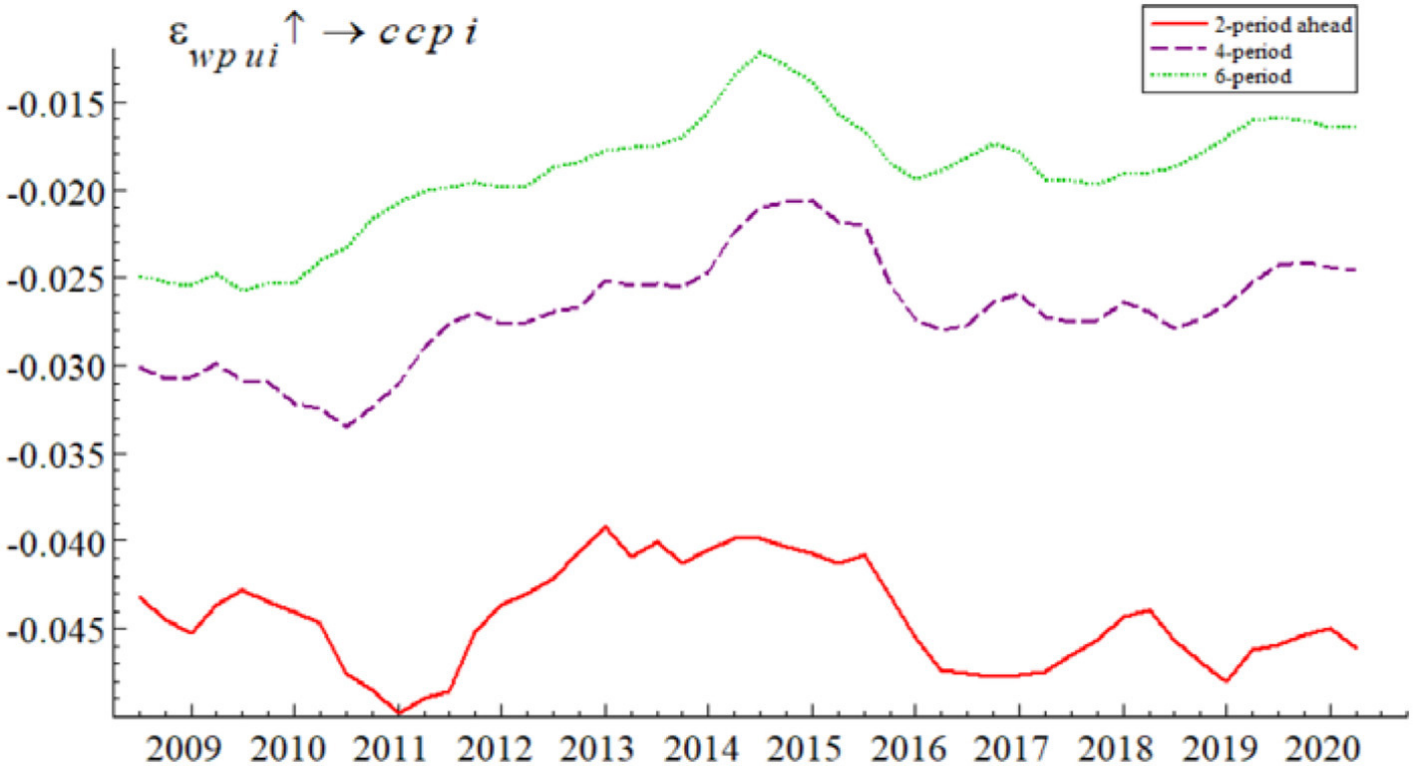
Time-varying effects of WPU on China's commodity prices. The red line represents the short-term impact, the purple line the medium-term impact and the green line the long-term impact.

As can be seen from Figure 3, WPU has a significant time-varying impact on China's commodity prices, and the effect of short-term WPU shocks on China's commodity prices is stronger than in the medium to long term. Specifically, given a one-unit positive shock to the WPU, the impulse response volatility of China's commodity prices with a 2-period lag is (−0.05, −0.04); the impulse response volatility of China's commodity prices with a 4-period lag is (−0.034, −0.022); and the impulse response volatility of China's commodity prices with a 6-period lag is (−0.025, −0.013).

The comparison results show that the short-term impact of WPU on China's commodity prices is greater than the medium and long-term impact. It is mainly because the financial attribute of commodities plays a rapid role in the short term. On the one hand, through the risk premium channel, the rise in WPU gives rise to systemic risks in the commodity futures market. The systemic risks will continue to accumulate and lead to the increase in the risk premium of commodity prices. On the other hand, through the futures investment channel, the rise in WPU leads to an increase in risk aversion among commodity futures investors. They then opt to withdraw from the commodity futures market to avoid risks. The reduction in commodity futures investment leads to the fluctuation of the commodity price. Therefore, WPU has a large negative impact on commodity prices in the short term. But in the long term, the negative impact decreases as the risk premium gradually increases.

The overall trend of the impulse response results in Figure 3 shows that the short- and medium-run effects of WPU on China's commodity prices are negative. This is because in the short run WPU shocks negatively affect commodity prices through the futures investment channel, in the medium and long run WPU shocks trigger lower commodity investment and demand through the corporate investment channel and the demand channel, which in turn leads to lower commodity prices. In addition, we find that the negative effect of the WPU on China's commodity prices during the 2014-early 2015 period was small, probably due to the fact that the China stock market was in a “bull market” phase in early 2014–2015, and the high stock prices drove up commodity prices with financial attributes, partially offsetting the negative effect of the WPU on China's commodity prices. The empirical results in this section verify the research hypothesis H1, and also confirm the views of Bakas and Triantafyllou (8) and Ezeaku et al. (51).

### Impact of WPU on China's commodity prices in different periods

In order to study whether the impact of WPU on China's commodity prices varies at different time points, this paper referred to the research of Balcilar et al. (24). We reviewed the major events in China in the sample period and selected three representative time periods, namely, the global financial crisis in the fourth quarter of 2008, the Chinese stock market crash in the second quarter of 2015 and the COVID-19 in the fourth quarter of 2019. We then compared and analyzed at which time point WPU had the greatest impact on China's commodity prices. The global financial crisis broke out in the fourth quarter of 2008 and quickly spread to China, leading to a recession in China real economy and triggering a decline in demand for commodities in China, reflecting the commodity attributes of commodities. The China stock market experienced a surge and a plunge in the second quarter of 2015, and the rise and fall of the China stock market triggered a different proportion of investors in the China commodity futures market, leading to a change in the degree of financialization of China commodities, reflecting the financial attributes of commodities. The outbreak of the COVID-19 epidemic in the fourth quarter of 2019 and the massive shutdown and suspension of production led to a recession and rising unemployment in China and increased uncertainty for the China real economy and financial markets.

**Figure 4:**
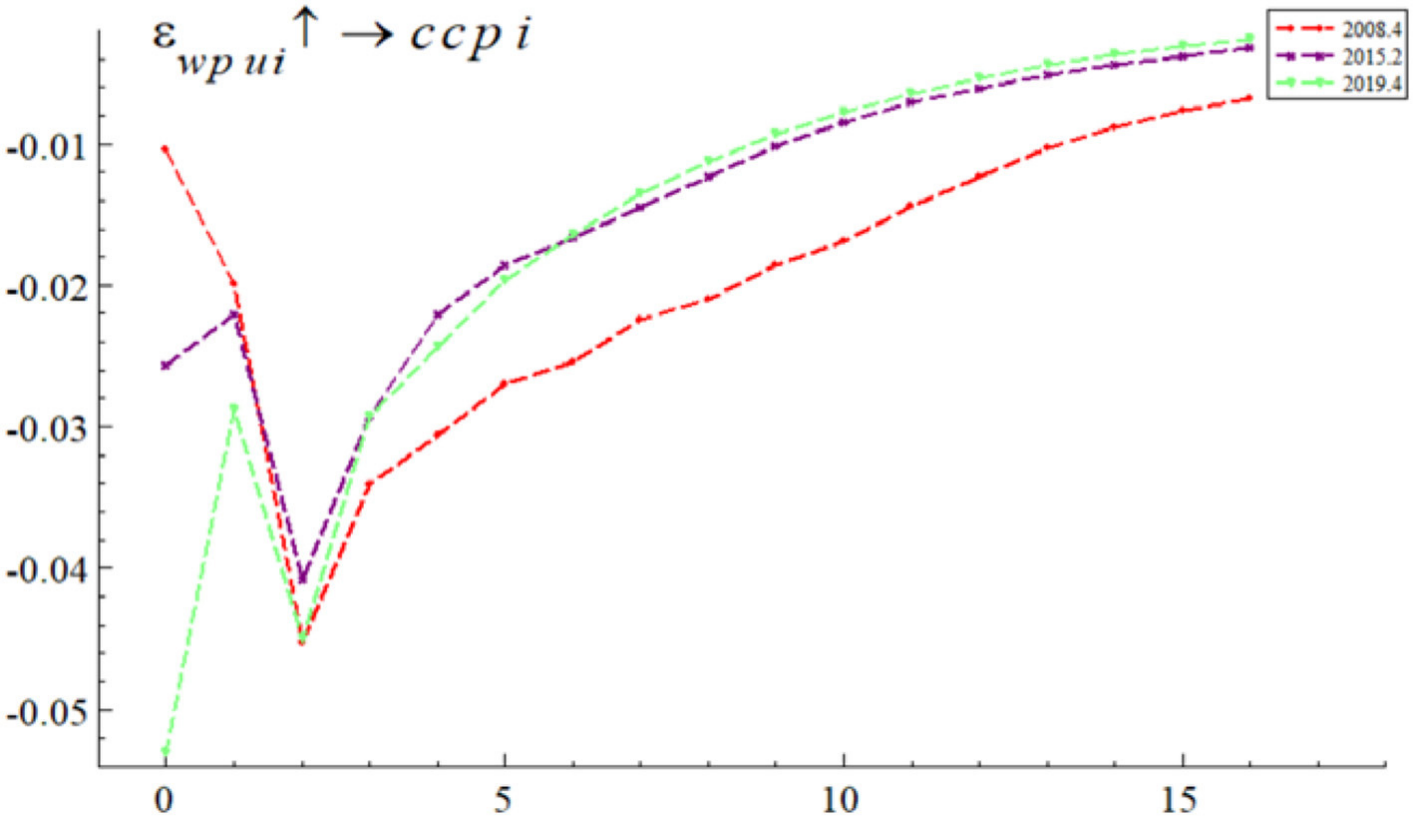
The effect of WPU on China's commodity prices at different points in time. The red line represents the fourth quarter of 2008, the purple line the second quarter of 2015, and the green line the fourth quarter of 2019.

As can be seen from Figure 4, given a one-unit positive shock to WPU, the impulse responses of China's commodity prices at these points in time were all negatively affected because WPU affected commodity prices *via* futures investment channel, corporate investment channel, and demand channel. However, the impacts were not exactly the same at each point in time. In terms of impact effect size, the WPU based on the fourth quarter of 2019 had the largest negative impact on China's commodity prices, followed by that in the second quarter of 2015 and that in the fourth quarter of 2008, with initial impulse response values of −0.054, −0.026, and −0.01, respectively. In terms of the speed of convergence of the impulse results, the impulse response results for all three time points converge from lag 2, but the impulse response results for the fourth quarter of 2019 converge the fastest, the impulse response results for the second quarter of 2015 converge the second fastest, and the impulse response results for the fourth quarter of 2008 converge the slowest.

Overall, the WPU shock during the COVID-19 epidemic had the largest negative impact on China's commodity prices. The WPU during the global financial crisis had the smallest negative impact on China's commodity prices, but the longest duration. WPU has different effects on China's commodity prices in different periods. The empirical results in this section verify the research hypothesis H2. They also confirm the finding of Long and Guo (52) that WPU has the greatest and significant impact on commodity prices at the time of COVID-19.

### Impact of WPU on commodity prices in different categories

To explore the impact of WPU on the prices of different categories of commodities, we selected minerals, non-ferrous metals, energy and steel, the four categories with the highest proportion in China's commodity market, and analyzed the impact of WPU on the prices of each category. These four types of commodities have large import volumes, are highly dependent on foreign countries, and with low industrial concentration in China, all of which render them subject to external factors. Previous research also suggests that during the pandemic the price index of these four types have more violent changes than other types, and that their prices are prone to resonating, which is likely to cause systemic financial risks. It is of great significance to study their responses to the shock of the pandemic.

**Figure 5:**
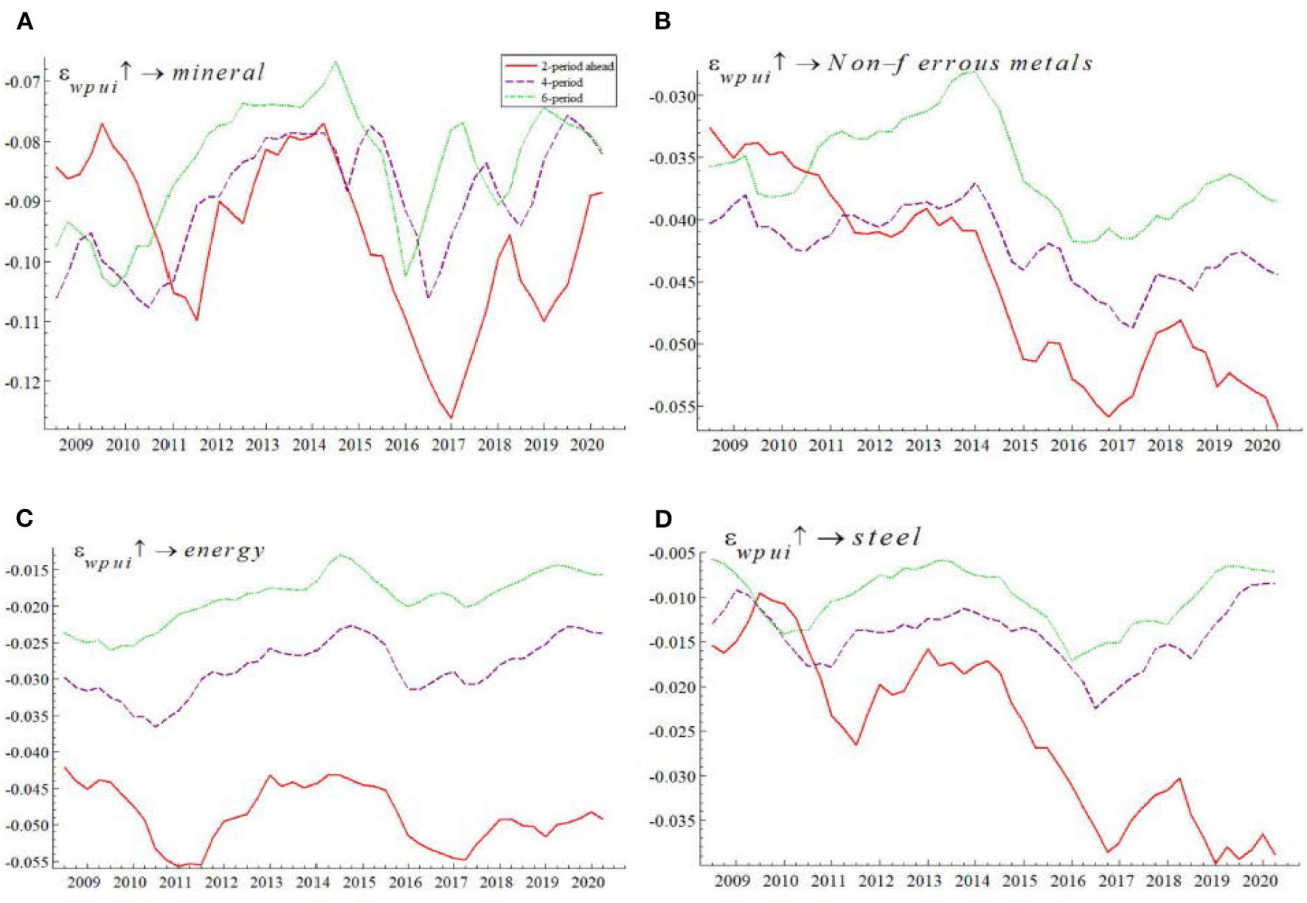
Time-varying effects of WPU on the prices of different categories of commodities (i.e., **(A)** shows mineral, **(B)** shows non-ferrous metals, **(C)** shows energy, and **(D)** shows steel).

As can be seen from Figure 5, WPU had a significantly negative time-varying effect on commodity prices across all categories, and the impact was stronger in the short term than in the medium and long term. This is consistent with the empirical results in Figure 3, indicating the robustness of the results.

Specifically, given a positive shock to the WPU unit, the impulse response interval was (−0.13, −0.068), (−0.04, −0.005) for steel, (−0.055, −0.015) for energy, and (−0.057, −0.028) for non-ferrous. The comparison results show that the time-varying effects of WPU on the commodity prices of minerals, non-ferrous metals, energy and steel metals are differentiated, and the WPU has the largest time-varying effects on the commodity prices of minerals and the smallest time-varying effects on the commodity prices of steel. The empirical results verify the research hypothesis H3, and is consistent to Xiao et al. (53) finding as the impact of steel stands at 0.012%, suggesting that compared to other commodities, steel is the least affected by the epidemic.

**Figure 6:**
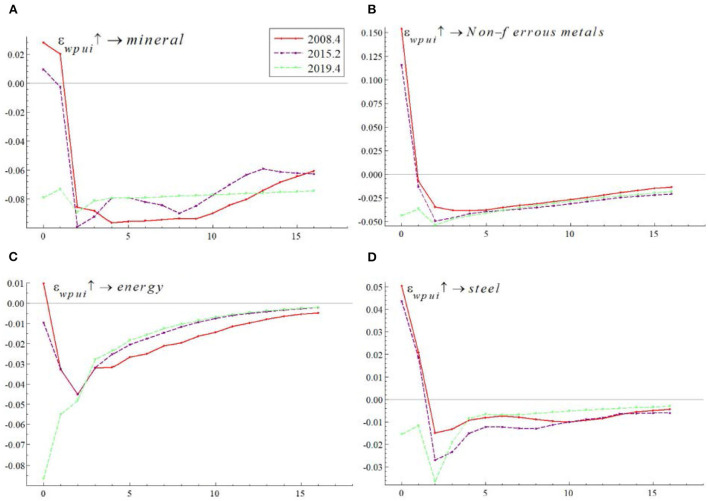
Impact of WPU on the prices of different categories of commodities (i.e., **(A)** shows mineral, **(B)** shows non-ferrous metals, **(C)** shows energy, and **(D)** shows steel).

Figure 6 documents the impact of WPU on commodity prices for minerals, non-ferrous metals, energy and steel at different points in time. By comparing the impulse response results for a single category of commodities at different points in time, we can find that the impact of the WPU shock differed at different points in time, which is consistent with the results in Figure 4. In addition, by comparing the impulse response results of mineral, steel, energy and non-ferrous metals commodities at the same time point, we find that there are large differences in the magnitude of the impulse response result values and the speed of convergence of the impulse response results, indicating the variability of the WPU effects on the prices of mineral, steel, energy and non-ferrous metals commodities.

## Conclusion and policy implication

Using the TVP-SVAR-SV model, we have explored the time-varying impact of WPU on Chinese commodity prices from the first quarter of 2008 to the second quarter of 2020. Specifically, we select minerals, non-ferrous metals, energy and steel commodities for a categorical study to compare and analyze the differences in the impact of WPU on the four categories of commodity prices.

The results are as follows. First, WPU has a significantly negative time-varying impact on China's commodity prices, and its effect was stronger in the short term than in the long term. Secondly, the WPU impact during the COVID-19 pandemic in the fourth quarter of 2019 is greater than during the global financial crisis in the fourth quarter of 2008 and during the China stock market crash in the second quarter of 2015. Third, the effect of WPU on commodity prices varies significantly across the four major categories, where it effects a greater time-varying impact on mineral prices than on the prices of steel.

Hence, we put forward the following policy implications to both the government and investors. First, government departments should adopt flexible policies to prevent systemic risks from spreading to the whole financial market in the short term, and reduce the impact of worries over the pandemic. Especial attention should be paid to the fluctuation in the price of mineral commodities. If necessary, price protection policies can be adopted to prevent the possible systematic risk caused by the sharp rebound of the price of such commodities soon after the pandemic. Second, investors are advised to dynamically adjust their portfolios during different periods of major events. For instance, during the COVID-19 pandemic, they could choose assets with less negative impact, such as steel and non-ferrous metals, while adopting risk management strategies to prevent the violent response of commodity spots or futures positions to the uncertainty of the world pandemic.

## Data availability statement

The datasets presented in this study can be found in online repositories. The names of the repository/repositories and accession number(s) can be found below: https://github.com/caoqiangsh/WPUI_CCPI/blob/main/Qwpui_gdp_cpi_i_fin_ccpi.xls.

## Author contributions

HC: writing and reviewing the manuscript. WY: data collection. X-qY: methodology. QC: writing the manuscript and estimations. All authors contributed to the article and approved the submitted version.

## Funding

We thank the following funds for their support: (1) 2022 Annual Bengbu Think Tank Construction and Social Science Planning Project, Optimal Path and Policy Research of Green Finance on Bengbu City's Double Carbon Goals (No. BB22C005). (2) Humanities Research Project of Anhui Provincial Education Department, Research on the Long-term Mechanism of New Rural Cooperative Finance for High-Quality Service to Common Wealth (No. SK2021A0277). (3) Graduate Research Innovation Fund Project of Anhui University of Finance and Economics (ACYC2020133).

## Conflict of interest

The authors declare that the research was conducted in the absence of any commercial or financial relationships that could be construed as a potential conflict of interest.

## Publisher's note

All claims expressed in this article are solely those of the authors and do not necessarily represent those of their affiliated organizations, or those of the publisher, the editors and the reviewers. Any product that may be evaluated in this article, or claim that may be made by its manufacturer, is not guaranteed or endorsed by the publisher.

## Footnotes

^1^*The WPU Index* is downloaded from https://worlduncertaintyindex.com/.

## References

[1] Ahir H Bloom N Furceri D. The World Uncertainty Index. SIEPR Working Paper No19-027,Stanford Institute for Economic Policy Research. Palo Alto, CA: Stanford University (2019).

[2] Goodell JW. COVID-19 and finance: agendas for future research. Fin Res Letters. (2020) 1:1–17. 10.1016/j.frl.2020.101512 32562472 PMC7152896

[3] Pak A Adegboye OA Adekunle AI Rahman KM McBryde ES Eisen DP. Economic consequences of the COVID-19 outbreak: the need for epidemic preparedness. Front Public Health. (2020) 1:241. 10.3389/fpubh.2020.00241 32574307 PMC7273352

[4] Baker SR Bloom N Davis SJ Stephen J Terry NWP. COVID-Induced Economic Uncertainty. NBER Working Paper NO. 26983 (2020).

[5] Ma C Rogers J Zhou S. Modern Pandemics: Recession Recovery. Cambridge: National Bureau of Economic Research (2020). Available online at: https://papers.ssrn.com/sol3/papers.cfm?abstract_id=3565646

[6] Jin C. Impact of the COVID-19 pandemic on China's stock market volatility, during and after the outbreak: evidence from an aRDL approach. Front Public Health. (2022) 1:810102. 10.3389/fpubh.2022.810102 35664100 PMC9159152

[7] Ji Q Zhang D Zhao Y. Searching for safe-haven assets during the COVID-19 pandemic. International Review of Financial Analysis. (2020) 1:1–13. 10.1016/j.irfa.2020.101526 PMC7244450 38620286

[8] Bakas D Triantafyllou A. Commodity price volatility and the economic uncertainty of pandemics. Econ Lett. (2020) 1:1–5. 10.1016/j.econlet.2020.109283

[9] Troster B Kublbock K. Unprecedented but not unpredictable: effects of the COVID-19 crisis on commodity-dependent countries. Eur J Dev Res. (2020) 5:1430–49. 10.1057/s41287-020-00313-9 33100597 PMC7575855

[10] World Bank. A Shock Like No Other: The Impact of COVID-19 on Commodity Markets. Washington, DC: World Bank (2020). Available online at: https://thedocs.worldbank.org/en/doc/558261587395154178-0050022020/original/CMOApril2020SpecialFocus1.pdf

[11] Zaremba A Kizys R Aharon DY Umar Z. Term spreads and the COVID-19 pandemic: evidence from international sovereign bond markets. Finance Res Lett. (2022) 1:1–16. 10.1016/j.frl.2021.102042 35013673 PMC8733935

[12] Ashraf BN. Stock markets' reaction to COVID-19: cases or fatalities? Res Int Bus Finance. (2020) 1:101249. 10.1016/j.ribaf.2020.101249 34170989 PMC7244441

[13] Feng G-F Yang H-C Gong Q Chang C-P. What is the exchange rate volatility response to COVID-19 and government interventions? Econ Anal Policy. (2021) 1:705–19. 10.1016/j.eap.2021.01.018

[14] Adekoya OB Oliyide JA. How COVID-19 drives connectedness among commodity and financial markets: evidence from TVP-VAR and causality-in-quantiles techniques. Resour Policy. (2021) 70:10898. 10.1016/j.resourpol.2020.101898 34173426 PMC7572357

[15] Borgards O Czudaj RL Hoang THV. Price overreactions in the commodity futures market: an intraday analysis of the Covid-19 pandemic impact. Resour Policy. (2021) 1:1–36. 10.1016/j.resourpol.2020.101966 PMC9759686 36569184

[16] Umar Z Riaz Y Zaremba A. Patterns of spillover in energy, agricultural, and metal markets: a connectedness analysis for years 1780-2020. Finance Res Lett. (2021) 1:1–7. 10.1016/j.frl.2021.101999

[17] Umar Z Gubareva M Naeem M Akhter A. Return and volatility transmission between oil price shocks and agricultural commodities. PLoS ONE. (2021) 2:e0246886. 10.1371/journal.pone.0246886 33606770 PMC7894939

[18] Galan-Gutierrez JA Martin-Garcia R. Fundamentals vs. financialization during extreme events: from backwardation to Contango, a copper market analysis during the COVID-19 pandemic mathematics. MDPI. (2022) 4:1–23. 10.3390/math10040559

[19] Aharon DY Umar Z Aziz MIA Xuan Vinh V. COVID-19 related media sentiment and the yield curve of G-7 economies. North Am J Econ Finance. (2022) 1:1–15. 10.1016/j.najef.2022.101678

[20] Esparcia C Jareno F Umar Z. Revisiting the safe haven role of Gold across time and frequencies during the COVID-19 pandemic. North Am J Econ Finance. (2022) 1:1–43. 10.1016/j.najef.2022.101677

[21] Dhaene G Sercu P Wu J. Volatility spillovers: a sparse multivariate GARCH approach with an application to commodity markets. J Futures Mark. (2022) 5:868–87. 10.1002/fut.22312

[22] Lin B Xu B. How to effectively stabilize China's commodity price fluctuations? Energy Econ. (2019) 84:104544. 10.1016/j.eneco.2019.104544

[23] Gozgor G Kablamaci B. The linkage between oil and agricultural commodity prices in the light of the perceived global risk. Agric Econ Zemedelska Ekonomika. (2014) 7:332–42. 10.17221/183/2013-AGRICECON

[24] Balcilar M Gabauer D Umar Z. Crude oil futures contracts and commodity markets: new evidence from a TVP-VAR extended joint connectedness approach. Resour Policy. (2021) 1:1–14. 10.1016/j.resourpol.2021.102219

[25] Umar Z Jareno F Escribano A. Dynamic return and volatility connectedness for dominant agricultural commodity markets during the COVID-19 pandemic era. Appl Econ. (2022) 9:1030–54. 10.1080/00036846.2021.1973949

[26] Umar Z Jareno F Escribano A. Oil price shocks and the return and volatility spillover between industrial and precious metals star. Energy Econ. (2021) 1:1–13. 10.1016/j.eneco.2021.105291

[27] Gozgor G Lau CKM Sheng X Yarovaya L. The role of uncertainty measures on the returns of gold. Econ Lett. (2019) 1:e108680. 10.1016/j.econlet.2019.108680

[28] Wei Y Wang Z Li D Chen X. Can infectious disease pandemic impact the long-term volatility and correlation of gold and crude oil markets? Finance Res Lett. (2022) 47:102648. 10.1016/j.frl.2021.102648

[29] Azimli A. Degree and structure of return dependence among commodities, energy stocks and international equity markets during the post-COVID-19 period. Resour Policy. (2022) 1:1–17. 10.1016/j.resourpol.2022.102679 35340262 PMC8935323

[30] Ngo Thai H. Oil prices and agricultural commodity markets: evidence from pre and during COVID-19 outbreak. Resour Policy. (2021) 73:102236. 10.1016/j.resourpol.2021.102236 34539035 PMC8434820

[31] Umar Z Zaremba A Olson D. Seven centuries of commodity co-movement: a wavelet analysis approach. Appl Econ Lett. (2022) 4:355–9. 10.1080/13504851.2020.1869151

[32] Chen P. Global oil prices, macroeconomic fundamentals and China's commodity sector comovements. Energy Policy. (2015) 1:284–94. 10.1016/j.enpol.2015.09.024

[33] Jin X Zhu F. Global oil shocks and China's commodity markets: the role of OVX. Emerg Mark Finance Trade. (2021) 3:914–29. 10.1080/1540496X.2019.1658075

[34] Tao C Diao G Cheng B. The dynamic impacts of the COVID-19 pandemic on log prices in China: an analysis based on the TVP-VAR model. Forests. (2021) 4:1–18. 10.3390/f12040449

[35] Prokopczuk M Stancu A Symeonidis L. The economic drivers of commodity market volatility. J Int Money Finance. (2019) 1:1–66. 10.1016/j.jimonfin.2019.102063

[36] Bloom N Floetotto M Jaimovich N Saporta-Eksten I Terry SJ. Really uncertain business cycles. Econometrica. (2018) 3:1031–65. 10.3982/ECTA10927

[37] Bakas D Triantafyllou A. The impact of uncertainty shocks on the volatility of commodity prices. J Int Money Finance. (2018) 1:96–111. 10.1016/j.jimonfin.2018.06.001

[38] Hamilton JD Wu JC. Risk premia in crude oil futures prices. J Int Money Finance. (2014) 1:9–37. 10.1016/j.jimonfin.2013.08.003

[39] Bloom N. Fluctuations in uncertainty. J Econ Perspect. (2014) 2:153–75. 10.1257/jep.28.2.153

[40] Kilian L. Not all oil price shocks are alike:disentangling demand and supply shocks in the crude oil. Am Econ Rev. (2009) 3:1053–69. 10.1257/aer.99.3.1053

[41] Bernanke BS. Irreversibility, Uncertainty, and Cyclical Investment. NBER Working Paper NO 0502. Cambridge: National Bureau of Economic Research (1980).

[42] Christiano LJ Motto R Rostagno M. Risk shocks. Am Econ Rev. (2014) 1:27–65. 10.1257/aer.104.1.27

[43] Huang JB Li YL Zhang HW Chen JY. The effects of uncertainty measures on commodity prices from a time-varying perspective. Int Rev Econ Finance. (2021) 1:100–14. 10.1016/j.iref.2020.09.001

[44] Zhang CG Chen XQ. The impact of global oil price shocks on China's bulk commodity markets and fundamental industries. Energy Policy. (2014) 1:32–41. 10.1016/j.enpol.2013.09.067

[45] Nakajima J. Time-varying parameter VAR model with stochastic volatility: an overview of methodology and empirical applications. Monetary Econ Stud. (2011) 1:107–42.

[46] Primiceri GE. Time varying structural vector autoregressions and monetary policy. Rev Econ Stud. (2005) 3:821–52. 10.1111/j.1467-937X.2005.00353.x

[47] Gozgor G Demir E Belas J Yesilyurt S. Does economic uncertainty affect domestic credits? An empirical investigation. J Int Finan Mark Instit Money. (2019) 63:101147. 10.1016/j.intfin.2019.101147

[48] Jacks DS Stuermer M. What drives commodity price booms and busts? Energy Econ. (2020) 85:104035. 10.1016/j.eneco.2018.05.023

[49] Frankel JA. Effects of speculation and interest rates in a “carry trade” model of commodity prices. J Int Money Finance. (2014) 1:88–112. 10.1016/j.jimonfin.2013.08.006

[50] Liu P Vedenov D Power GJ. Commodity financialization and sector ETFs: evidence from crude oil futures. Res Int Bus Finance. (2020) 51:101109. 10.1016/j.ribaf.2019.101109

[51] Ezeaku HC Asongu SA Nnanna J. Volatility of international commodity prices in times of COVID-19: effects of oil supply and global demand shocks. Extract Indust Society Int J. (2021) 1:257–70. 10.1016/j.exis.2020.12.013

[52] Long S Guo J. Infectious disease equity market volatility, geopolitical risk, speculation, and commodity returns: comparative analysis of five epidemic outbreaks. Res Int Bus Finance. (2022) 1:101689. 10.1016/j.ribaf.2022.101689 35662835 PMC9150896

[53] Xiao D Su J Ayub B. Economic policy uncertainty and commodity market volatility: implications for economic recovery. Environ Sci Pollut Res. (2022) 1:1–12. 10.1007/s11356-022-19328-2 35426558 PMC9010710

